# An Inducible hiPSC-Derived Human Podocyte Model for Functional Analysis of TRPC6 Variants Associated with FSGS

**DOI:** 10.3390/cells15080712

**Published:** 2026-04-17

**Authors:** Lilas Batool, Krithika Hariharan, Gabriel Stölting, Tingting Zhong, Dimitry Tsvetkov, Manfred Gossen, Andreas Kurtz

**Affiliations:** 1 Hamburg Center for Kidney Health (HCKH), University Medical Center Hamburg-Eppendorf, 20251 Hamburg, Germany; l.batool@uke.de; 2 Berlin Institute of Health (BIH), Charité Medical University Berlin, 10117 Berlin, Germany; dr.krithika.hariharan@gmail.com (K.H.); tingting.zhong@charite.de (T.Z.); manfred.gossen@charite.de (M.G.); 3 Center of Genomic Medicine, Berlin Institute of Health (BIH), Charité Medical University Berlin, 10117 Berlin, Germany; gabriel.stoelting@bih-charite.de; 4 Center for Internal Medicine, University Medical Center Hamburg-Eppendorf, 20251 Hamburg, Germany; d.tsvetkov@uke.de; 5 Institute of Active Polymers, Helmholtz-Zentrum Hereon, 14513 Teltow, Germany

**Keywords:** FSGS, *TRPC6*, hiPSC, podocytes

## Abstract

Podocyte injury is a characteristic feature of focal segmental glomerulosclerosis (FSGS) that leads to the development of nephrosis as its loss causes proteinuria and progressive glomerulosclerosis. The physiological function of podocytes is critically dependent on proper intracellular calcium levels; an excess or shortage of calcium influx in these cells may result in foot process effacement, apoptosis, and nephron degeneration. A key protein responsible for the regulation of calcium flux is the canonical transient receptor potential 6 (TRPC6) expressed in podocytes. Several mutations in the *TRPC6* gene have been associated with FSGS. Here we present a systematically optimized inducible FSGS model system in human induced pluripotent stem cells (hiPSCs). We generated and phenotypically characterized three transgenic hiPSC lines with regulatable overexpression of *TRPC6* wild-type and FSGS-associated gain-of-function (GoF, P112Q) and loss-of-function (LoF, G757D) mutations. Moreover, these cell lines were differentiated into induced podocytes (ipodocytes). We assessed the impact of *TRPC6* GoF and LoF mutants on calcium influx in combination with *TRPC6* agonists and antagonists. Our data showed relative calcium responses consistent with the GoF and LoF phenotypes. Transgenic iPSC-based models, like the one presented here, are instrumental to studying disease mechanisms in vitro and investigating the outcomes of, and possible therapeutic interventions for, this complex disease.

## 1. Introduction

Focal segmental glomerulosclerosis (FSGS) is a kidney disease characterized by focal and segmental sclerosis in glomeruli. The clinical manifestations of FSGS include marked proteinuria, hypertension, steroid resistance, and a high rate of progression to renal failure in children and adults [1]. Following kidney transplantation, 30–40% of FSGS patients experience recurrence, which presents as early nephrotic syndrome and the loss of foot processes that rapidly progress into dominant sclerosis [2,3]. The incidence of FSGS has increased up to eightfold during the past 20 years [4,5].

For normal glomerular function, the major components of the glomerular filter (endothelial cells, the glomerular basement membrane (GBM), and podocytes) must remain intact to maintain an effective filtration barrier. Podocyte foot processes and the glomerular slit diaphragm are the essential elements of the glomerular filter [6]. Many podocytopathies, glomerulopathies and some forms of FSGS, are characterized by podocyte foot process effacement, actin cytoskeletal remodeling, or the detachment of podocytes from the GBM. Data from animal models suggest that injury directed or originating within the podocyte constitutes a critical event that can lead to FSGS [7].

Human genetic studies have identified at least 27 genes involved in dominant or recessive forms of FSGS [8]. The majority of gene products are involved in the development, structural architecture, and function of podocytes. Heterozygous mutations in transient receptor potential cation channel, subfamily C, member 6 (*TRPC6*) are associated with late as well as early onset of FSGS. The transient receptor potential (TRP) family of calcium channels contains more than 50 members separated into seven subfamilies of channel subunits that perform a variety of cellular functions [9,10]. TRPCs facilitate an influx of calcium in response to phospholipase C (PLC)-mediated signals [11,12] and subsequent breakdown of phosphatidylinositides, with the generation of diacylglycerol (DAG) directly activating TRPC6 [13,14]. In the kidney, TRPC6 is abundant in the podocyte foot process and in the collecting duct. It was also found to be allied with nephrin and podocin, both essential protein components of the slit diaphragm [15]. The 25 known *TRPC6* mutations causing autosomal-dominant FSGS are exclusively missense mutations [15,16,17,18,19,20,21,22,23,24,25,26,27]. Notably, both gain-of-function (GoF) and loss-of-function (LoF) mutations in the TRPC6 channel are believed to evoke the FSGS clinical phenotype [22,28]. However, how these functionally distinct FSGS-associated TRPC6 mutants cause a similar phenotype is poorly understood.

In order to gain a deeper insight into podocytopathies, animal models and in vitro models using established cell lines have been investigated. However, animal models frequently produce results that are not directly applicable to humans [29]. Hence, there is a critical need for human models that replicate glomerular physiology and pathology to uncover the molecular mechanisms of underlying glomerular diseases. Previously, stable human cell lines, including podocyte lines, have been used to study aspects of podocyte physiology [30,31] Human-induced pluripotent stem cells (hiPSC) have extended the capabilities of personalized human cell models and their utilization in tissue engineering and regenerative medicine [29,32]. Multiple hiPSC-derived 2D podocytes and 3D kidney organoid models have been established [29,33,34]. We previously developed a protocol for the generation of hiPSC-derived podocytes by recapitulating the in vivo interactive cellular and signaling environment, making different developmental time points accessible for drug testing, organ modeling, and selective applications in regenerative medicine [35,36].

In this study, we developed an hiPSC-based model to study the effects of TRCP6 mutations on calcium influx in podocytes. Genetically modified hiPSC for controlled overexpression of the TRPC6 wild-type (BCRTi015-A-1) and the FSGS-associated the gain-of-function (GoF, BCRTi015-A-2) and loss-of-function (LoF, BCRTi015-A-3) mutations P112Q and G757D were differentiated into podocytes (ipodocytes). Calcium influx patterns showed relative calcium responses consistent with GoF and LoF genotypes. The presented induced TRPC6 (iTRPC6) lines provide the basis for generating multiple human cell types to understand the role of TRPC6 mutations in high-throughput, homogenous and large-scale assays [37].

## 2. Materials and Methods

### 2.1. Site-Directed Mutagenesis Using Tet-On 3G System

By adapting the established protocols [22], we utilized two TRPC6 variants (P112Q and G757D) and a wild-type control, each consisting of a C-terminal YFP fusion protein. To achieve controlled expression within the WISCi004-A hiPSC line, we used a Tet-On 3G induction system [38], where transgene transcription is modulated by doxycycline (Dox) concentration via a pTRE3G promoter. The TRPC6-YFP sequences were amplified by PCR from a template plasmid (Addgene #21084) using Q5 high-fidelity polymerase and specific primers (Supplementary Table S1) using 2× Taq Master mix (New England Biolabs, Ipseich, MA, USA) by applying a three-step thermocycling protocol 98 °C for 30 s (1× cycle), 98 °C for 10 s, 59 °C for 30 s, 72 °C for 30 s (30× cycle) and 72 °C for 2 min (1× cycle). The resulting amplicons were integrated into the multiple cloning site (MCS) of a linearized XLone-GFP all-in-one vector (Addgene #96930) via KpnI and BsrGI restriction digestion enzymes and a subsequent ligation step. Following transformation into *E. coli* using Top 10 F cells (ThermoFisher Scientific, Waltham, MA, USA), successful clones were validated by sequencing. High-purity plasmid DNA for downstream transfection was then isolated using Nucleobond Xtra Maxi Kits (Macherey-Nagel, Düren, Germany), following the manufacturer protocol. All procedures were performed according to the manufacturer’s instructions. All plasmids were sequence-verified by Sanger Sequencing through Microsynth Seqlab, Göttingen, Germany.

### 2.2. Gene Targeting Constructs and Molecular Cloning Using Dual Genomic Safe Harbor (GSH) Targeting Strategy

The CAMi014-A hiPSC line, derived from male dermal fibroblasts (A1ATD1), was a generous gift from the Kotter Laboratory at the University of Cambridge. This line features a constitutive expression cassette for the rtTA3G transactivator, driven by a CAG promoter and specifically targeting the ROSA26 genomic safe harbor locus to ensure stable and predictable induction [39].

The inducible TRPC6 pAAV_TRE-TRPC6-EGFP targeting vector was constructed by the ligation (Quick LigationTM Kit, New England BioLabs, Beverley, MA) of the TRPC6 gene sequence (PCR-amplified from the original plasmid addgene #21084) and EGFP gene sequence (PCR-amplified from pAAV_TRE-EGFP) into the EcoRI/SpeI sites of the pAAV_TRE-EGFP plasmid. The vector was constructed for each TRPC6 wild-type and mutants (pAAV_TRE-WT-EGFP, pAAV_TRE-P112Q-EGFP, and pAAV_TRE-G757D-EGFP). The pAAV_TRE-TRPC6-EGFP plasmids were transformed into E. coli Top 10 F (ThermoFisher Scientific) competent cells. Following the isolation of plasmid DNA from multiple clones, the constructs were validated via sequencing. We then utilized Nucleobond Xtra Maxi Kits to generate high-yield, transfection-grade DNA preparations, adhering to the protocols provided by the manufacturer. All plasmids were sequence verified by Sanger Sequencing through Microsynth Seqlab, Germany.

To integrate the Tet-ON-TRPC6-EGFP transgene in the AAVS1 locus, previous ROSA26 targeted A1ATD1 cell lines were seeded (0.15 × 106 cells/well) in feeder-free culture conditions in mTeSR^TM^1 (STEMCELL Technologies, Vancouver, BC, Canada) medium on Geltrex^TM^ (Life Technologies, Carlsbad, CA, USA)-coated 6-well plates and transfected 48 h after passaging. Transfection was performed in Opti-MEM^TM^ (Gibco, Grand Island, NY, USA) supplemented with Lipofectamine^TM^ 3000 (Invitrogen, Carlsbad, CA, USA) according to the manufacturer’s protocol. A total of 2 µg of plasmid DNA consisting of (i) 0.5 µg pZFN-AAVS1-L-ELD, (ii) 0.5 µg pZFN-AAVS1-R_KKR, and (iii) 1 µg PUC-AAV_TRE-TRPC6-EGFP was used per reaction. DNA–lipid complexes were added to the cells dropwise and incubated for 24 h. The next day, the medium was removed and replenished with mTESR1 medium. After 24 h, 1 µg/mL Puromycin (Sigma-Aldrich, St. Louis, MO, USA)was added to the culture medium. The selection was carried out for at least three days. The presence of the transgene was visualized in the wells with doxycycline (0.5 µg/mL). Individual colonies were picked and expanded for the following 7–10 days of selection and were analyzed by genotyping.

Homogenous cell lines were validated through genomic DNA isolation from a 6-well plate using the NucleoSpin® Tissue Genomic DNA Kit (Macherey-Nagel, Düren, Germany)). We adopted the genotyping strategy previously described by Pawlowski et al. [39] to confirm successful integration. Detailed information regarding the adapted PCR conditions and the specific primer sequences utilized in this study are provided in Supplementary Table S1.

### 2.3. Pluripotent Stem Cell Culture

The CAMi014-A (A1ATD1) cell line (between passage 39 and 60) was used in this study. The cells were maintained and expanded in a feeder-free environment using mTeSR^TM^1 (STEMCELL Technologies, Vancouver, BC, Canada) on a Geltrex-coated surface (Life Technologies, Carlsbad, CA, USA). Daily media change and passaging every 3–5 days upon reaching confluence was performed by incubating cells with 0.5 mM EDTA (ThermoFisher Scientific, Waltham, MA, USA) for 5 min at room temperature, followed by mechanical detachment. The resulting cellular aggregates were re-plated at a split ratio of 1:6.

### 2.4. Podocyte and Proximal Tubular Epithelial Cell (PTEC) Differentiation

Podocyte differentiation was adapted from a protocol published by Hariharan et al. [40]. Briefly, iTRPC6 hiPSC lines at 70–80% confluence was dissociated into single cells using Accutase® (Life Technologies, Carlsbad, CA, USA)) and plated at a density of 10,000 cells/cm^2^ on Geltrex-coated plates. Initial seeding was performed in mTeSR-1 medium supplemented with 10 µM Y27632 (Wako, Pure Chemical Industries, Osaka, Japan). Following a 48 h recovery period, differentiation was initiated through three distinct phases. Stage I (Days 0–3) involved intermediate mesoderm induction using STEMdiff^TM^ APEL2^TM^ medium (STEMCELL Technologies, Vancouver, BC, Canada) supplemented with Activin A (10 ng/mL), rhBMP-4 (30 ng/mL), all-trans-retinoic acid (1 µM), and 5% PFHM-II. To promote Stage II (Days 4–7) metanephric mesenchyme commitment, the medium was transitioned to include 150 ng/mL GDNF and 5% PFHM-II, with refreshments every 48 h. For terminal podocyte maturation (Stage III), day 8 progenitors were harvested and re-seeded at 8000 cells/cm^2^ on Laminin-521 (BioLamina, Sundbyberg, Sweden) in the presence of 50 ng/mL rhHGF. By day 14, the culture exhibited characteristic arborized morphologies and bi-nucleated phenotypes. Differentiation success was validated through stage-specific marker analysis at days 4, 8, and 14. Additionally, day 8 cells were matured in Renal Epithelial Growth Medium (REGM) at a density of 15,000 cells/cm^2^ resulting in a confluent, multi-layered epithelial population featuring absorptive brush border characteristics.

### 2.5. Cardiomyocyte (CM) Differentiation

Cardiomyocyte (CM) differentiation was performed using a biphasic Wnt signaling modulation protocol adapted from established protocol [41]. Initially, hiPSC-iTRPC6 lines at 80% confluence were dissociated into single cells and seeded at a density of 10,000–20,000 cells/cm^2^ in the presence of 10 µM Y27632 (Wako, Pure Chemical Industries, Osaka, Japan). After 24 h, the medium was changed to Essential 8^TM^ Medium (Thermo Fisher Scientific, Waltham, MA, USA)without inhibitor and refreshed daily until the cultures reached 70–80% confluence. Initial stage of differentiation (Day 0) was inducing via mesodermal lineage commitment through the activation of Wnt signaling and initiated by cardiac priming medium (6 μM CHIR 99021 in RPMI-1640 supplemented with B27 minus insulin) for 48 h. Subsequently, the cardiac lineage was specified by inhibiting Wnt signaling for two days using cardiac induction medium containing 5 μM IWP-2. From Day 4 to Day 6, cells were maintained in basal medium with daily medium change before switching to CM maintenance medium (RPMI-1640 plus 1× B-27 supplement). Spontaneous contractile activity was monitored every 24–48 h, with initial beating typically observed between days 7 and 14. Detailed media formulations are provided in Supplementary Table S2.

### 2.6. Immunofluorescence Staining of Cells

For immunofluorescence analysis, cells were rinsed with phosphate-buffered saline (PBS; Thermo Fisher Scientific, Waltham, MA, USA) and fixed for 10 min using Cytofix^TM^ (BD Biosciences, San Jose, CA, USA). Following fixation, cells were permeabilized with BD Perm/Wash for 15 min, washed twice with PBS, and blocked for 30 min at room temperature using 10% host-specific secondary antibody. Primary antibody incubation (Supplementary Table S3) was conducted overnight at 4 °C. After three subsequent washes, cells were incubated with fluorophore-conjugated secondary antibodies in washing buffer for 1 h at room temperature. Nuclei were counterstained with 4′,6-diamidino-2-phenylindole (DAPI; Sigma-Aldrich, St. Louis, MO, USA). High-content imaging was performed using the Operetta and Opera Phenix systems (PerkinElmer, Waltham, MA, USA), with data analysis via Columbus software (version 2.4, PerkinElmer, Waltham, MA, USA). All assays were performed in triplicate across three independent biological replicates.

### 2.7. Flow Cytometry

Following dissociation with Trypsin/EDTA (PAN Biotech, Aidenbach, Germany), cells were labeled with LIVE/DEAD^TM^ Fixable Blue for 30 min. GFP expression was induced with doxycycline 24 h before FACS processing. For intracellular markers, cells were permeabilized for 15 min in Phosflow Perm Buffer II (BD Biosciences, San Jose, CA, USA). Primary antibody staining was performed in ice-cold DPBS containing 2% FCS for 30 min, followed by secondary antibody labeling. Flow cytometric data were collected using a MACSQuant® VYB system (Miltenyi Biotec, Bergisch Gladbach, Germany) and analyzed via FlowJo^TM^ software (version 10.8.1, FlowJo LLC, Ashland, OR, USA). All reported data represent at least three biological and three technical replicates per cell line.

### 2.8. Quantitative Real-Time PCR (qPCR)

RNA was extracted from each mutant cell line, using the RNeasy® Mini Kit (QIAGEN, Hilden, Germany)). DNAse I, an RNase-free kit (Thermo Fisher Scientific, Vilnius, Lithuania; catalog no. EN052) was used to remove contaminating DNA. cDNA was generated with TaqMan^TM^ Reverse Transcription Reagents (Thermo Fisher Scientific, Waltham, MA, USA) and qPCR was done on QuantStudioTM 6 Flex Real-Time PCR system using SYBR Green PCR Master Mix (Applied Biosystems, Foster City, CA, USA). Each reaction was performed with three technical replicates, and *GAPDH* served as the internal reference control. Data were analyzed according to the ΔΔCt comparative threshold method.

### 2.9. Albumin Assay

To evaluate podocyte permeability and endocytic capacity, cells were differentiated for 14 days following established protocol [40]. Post-differentiation, the maintenance medium was substituted with serum-free media containing FITC-labeled albumin (0.5 mg/mL, Abcam, Cambridge, UK). After 1 h incubation at 37 °C, nuclei were counterstained using Hoechst 33342. Live-cell imaging was conducted on the Opera Phenix system to quantify albumin. Subsequently, the samples were fixed in 4% paraformaldehyde (PFA). All permeability assays were executed in triplicate to ensure statistical robustness.

### 2.10. Calcium Imaging of Podocyte

For calcium imaging, day 8 differentiated cells were re-seeded onto Laminin-521-coated, 13 mm cover slips for terminal maturation. Dox was administered on day 12 to observe the ipodocytes expressing iTRCP6-EGFP at day 14. Prior to imaging, cells were rinsed with PBS and loaded with Fura-2 AM (2 µM) for 30 min at 37 °C in the dark. Coverslips were then transferred to a recording chamber under continuous perfusion with HEPES-buffered physiological saline (134 µM NaCl, 6 µM KCl, 1 µM MgCl_2,_ 1 µM CaCl_2_, 10 µM glucose, and 10 mM HEPES; pH 7.4). For agonist-induced responses, a 15 min recording was performed, consisting of a 3 min baseline, 2 min of Carbachol or DOG (100 µM), and a final 10 min wash. To evaluate TRPC6 inhibition, cells were pre-incubated with SAR7334 (100 µM) for 10 min prior to a 5 min co-application of the agonist and inhibitor. Ratiometric fluorescence was captured by alternating excitations at 340 and 385 nm using a FuraLED source and an OptiMOS camera (OptiMOS^TM^ camera (ANDOR, Belfast, Northern Ireland, UK; 100 ms intervals, 10 ms exposure). EGFP-positive cells were manually selected as regions of interest (ROIs) in Fiji, and calcium transients were processed using a custom Python script (Python, version 3.9, Python Software Foundation, Wilmington, DE, USA).

### 2.11. Immunoblotting

RIPA buffer (Sigma) containing 1× protease inhibitor (Roche, Basel, Switzerland) was used to extract protein from cells. The resulting lysates were sonicated for 2 min and clarified by centrifugation at 16,000× *g* for 20 min. Protein concentration in the supernatant was determined using the BCA Protein Assay Kit (ThermoFisher Scientific, Waltham, MA, USA). Samples were prepared by combining 10 µg of protein with 2× Laemili Buffer (BioRad, Laboratories, Hercules, CA, USA), and 100 mM Dithioreitol (DTT; ThermoFisher, Waltham, MA, USA) to a final volume of 30 µL. Following denaturation at 70 °C for 10 min, proteins were resolved on 12% Bis-Tris mini gels (Invitrogen, Calsbad, CA, USA) using MOPS SDS running buffer, with a 10–180 kDa PageRuler (ThermoFisher Scientific, Waltham, MA, USA) serving as the molecular weight standard. Proteins were transferred to 0.45 µm nitrocellulose membranes (GE Healthcare, Chicago, IL, USA) via wet blotting. After blocking with Intercept^®^ T20 (TBS) Diluent (LI-COR Biosciences, Lincoln, NE, USA)) for 1 h at room temperature, membranes were incubated overnight at 4 °C with primary antibodies against GFP (mouse, 1:5000), TRPC6 (rabbit, 1:300), and beta-actin (rabbit, 1:1000). The next day, after three 10 min washes (twice in TBS-T and once in TBS), membranes were incubated with IRDye-conjugated secondary antibodies (800 CW anti-mouse and 680 RD anti-rabbit; 1:10,000) for 1 h. Finally, protein bands were visualized using the ChemiDoc MP Imaging System (Bio-Rad, Hercules, CA, USA).

### 2.12. Transmission Electron Microscopy

For ultrastructural analysis, cell cultures were primary-fixed in 2.5% glutaraldehyde (Serva) buffered with 0.1 M sodium cacodylate for 30 min at room temperature, followed by storage at 4 °C. Secondary fixation was performed using 1% osmium tetroxide (Electron Microscopy Sciences, Hatfield, PA, USA) and 0.8% potassium ferrocyanide II (Carl Roth, Karlsruhe, Germany) in cacodylate buffer. The samples were subsequently stabilized by overnight embedding in agarose. After sectioning the agarose into blocks, the specimens underwent dehydration through a graded ethanol series and were infiltrated with Epon resin (Roth). Ultrathin sections (70 nm) were prepared and contrast-enhanced with uranyl acetate and lead citrate. Imaging was conducted using a Zeiss Leo 906 electron microscope (Carl Zeiss, Oberkochen, Germany) operating at an 80 kV acceleration voltage, with images captured via a 2 K slow-scan CCD camera (TRS, Moorenweis, Germany)

### 2.13. Scanning Electron Microscopy

For Scanning Electron Microscopy (SEM), cells seeded on coverslips were fixed in 2.5% glutaraldehyde (pH 7.3) buffered with cacodylate for 30 min at room temperature, followed by storage at 4 °C. Secondary fixation was achieved using 2% osmium tetroxide (EMS) in 0.1 M cacodylate for 90 min. Specimens subsequently underwent dehydration through ethanol gradient series and were processed using a critical point dryer (Leica Microsystem, Wetzlar, Germany). To ensure electrical conductivity, the samples were coated with a gold–palladium alloy via sputtering (Bal-Tec, Balzer, Liechtenstein) and maintained under vacuum. Ultrastructural examination was performed using a GeminiSEM 300 (Carl Zeiss) scanning electron microscope.

## 3. Results

### 3.1. Generation of hiPSCs with Inducible Expression of Wild-Type, GoF and LoF TRPC6

**Figure 1:**
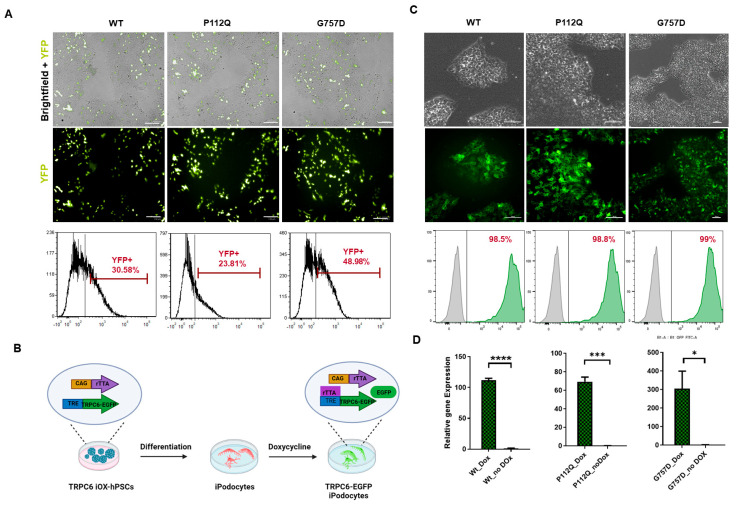
Generation of inducible TRPC6 expression system in hiPSCs. (**A**) Representative images illustrate the development of Tet-On all-in-one vector TRPC6 hiPSCs (WISCi004-A), expressing YFP after transfection, Blasticidin selection, and FACS sorting, as shown by the merged images of bright field and YFP signal for each variant under study. Scale bar 100 µm. Below the panel is a representative flow cytometry analysis, demonstrating a depleted population of up to 40% YFP-positive cells. (**B**) Developmental strategy for the conversion of TRPC6-EGFP hiPSCs into ipodocytes. (**C**) The generation of dual GSH-targeted (CAMi014-A)-inducible TRPC6-EGFP hPSCs cell lines WT, GoF (P112Q), LoF (G757D) in bright-field microscopy and the EGFP signals indicate TRPC6 expression. Scale bar 100 µm. The panel below is a representative flow cytometry analysis of transgenic hiPSCs, demonstrating an enriched population of up to 90% EGFP-positive cells after 24 h of doxycycline (Dox) treatment, shown in green, and negative control, shown in gray. (**D**) qPCR analysis for the relative TRPC6 expression of cells with (Dox) and without (no Dox) in wild-type (Wt), GoF (P112Q) and LoF (G757D) hiPSC lines.T test(and nonparametric tests) was performed indicating value (**** = *p* < 0.001, *** = *p* < 0.002, and * = *p* < 0.0371, with *n* = 3 technical replicates).

Tet-On single vector constructs, also known as “Tet-On-all-in-one” systems [38], have been designed to introduce the elements necessary for conditional gene expression, namely the reverse tetracycline-controlled transactivator (rtTA) and the Tet-promoter, in one single step in a predetermined arrangement. We used the hiPSC-line WISCi004-A to integrate *TRPC6* wild-type and mutant genes tagged with YFP for Tet-inducible expression. However, this system generated a mosaic transgenic cell population, despite blasticidin drug selection and fluorescence-activated cell sorting (FACS) (Figure 1A) followed by the silencing of transgene after two to three passages. Therefore, the system was substituted with a two-vector Tet system, and experiments in hiPSCs were conducted in the OPTi-OX^®^ cell line, a commercially available hiPSC line (CAMi014-A) that constitutively expresses rTA3G in the ROSA26 locus under the control of the CAG promoter [39].

The Tet-promoter–TRPC6-EGFP gene unit was transfected to the AAVS1 safe harbor locus of the CAMi014-A cells using zinc-finger nucleases (ZFN) (Figure 1B and Figure S1A). Isogenic cell lines were isolated, expanded, and genotyped. From the genotyped clones, a homozygous clone of each variant, i.e., wild-type, P112Q (GoF), and G757D (LoF), carrying two copies of each transgene and showing high transgene expression were selected for the experiments (Figure S1). The expression of TRPC6-EGFP variants was induced homogenously with doxycycline (Dox), which remained consistent across multiple clones and passages, with >90% of cells expressing TRPC6-EGFP as observed by FACS analysis (Figure 1C). Each of the established inducible TRPC6- (iTRPC6) cell lines (BCRTi015-A-1, designated as iTRPC6-wt; BCRTi015-A-2, designated as iTRPC6-GoF; and BCRTi015-A-3, designated as iTRPC6-LoF) expressed pluripotency markers (Figure S1C). TRPC6 gene expression was quantified and confirmed by real-time quantitative PCR (qPCR) in the presence and/or absence of Dox (Figure 1D).

### 3.2. Differentiation of iTRPC6 Cell Lines Towards Renal Cell Types

**Figure 2:**
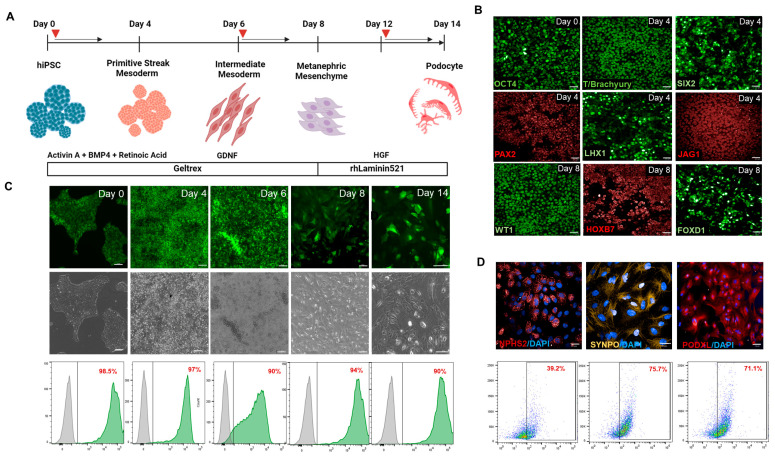
Directed differentiation of hiPSCs iTRPC6-EGFP into podocytes. (**A**) Schematic overview of the protocol for the derivation of mature glomerular podocytes. Red arrows show induction of Doxycycline at different differential stages (**B**) Subsequent appearance of transcription factors at different stages of the differentiation protocol; immunofluorescence staining for renal vesicle developmental stages indicates OCT4-expressing cells (day 0), and the expression of intermediate mesoderm and metanephric mesenchyme markers PAX2, SIX2, LHX1 by day 4 and ureteric progenitor marker HOXB7 and FOXD1 by day 8. Scale bar = 100 µm. (**C**) Morphological changes in hiPSCs at each stage of differentiation. The lower panel shows a representative flow cytometry analysis of transgenic iPSCs demonstrating iTRPC6-EGFP expression on differentiation days 0, 4, 6, 8, (scale bar = 100 µm) and 14 (Scale bar = 25 µm). Gray population (left side) indicates un-transfected cells while green population (right side indicates TRPC6 tagged GFP expression (**D**) Immunofluorescence staining of podocyte markers—NPHS2, SYNPO, and PODXL. Scale bar = 25 µm. The lower panel shows a representative flow cytometry analysis of podocyte markers—NPHS2, SYNPO, and PODXL.

The established inducible *TRPC6* (iTRPC6-wt, iTRPC6-GoF, iTRPC6-LoF) lines were differentiated into podocytes using an established protocol [40] (Figure 2A). iTRPC6 lines were directed towards intermediate mesoderm (IM) cells by using APEL differentiation medium containing Activin A, BMP4, and Retinoic acid that increased SIX2, PAX2, and WT1 expression (Figure 2B). The specification of IM and nephrogenesis was further spurred by the addition of GDNF, as shown, to promote ureteric epithelium differentiation, and ureteric branching provides positive feedback for metanephric mesenchyme cells [42,43]. Completion of the 8-day treatment resulted in the expression of WT1, HOXB7 and JAG1 (Figure 2B) confirming progression to renal vesicle cells. Day 8 renal vesicle cells were harvested, and plated at low density on Laminin-521, in the presence of HGF, in APEL medium. On day 14, all iTRPC6 variant-derived cells acquired an arborized cell body with multi-nucleated podocyte-like phenotype (Figure 2C). To test the applicability of the utilized genomic safe harbor (GSH) platform for the induction of TRPC6 transgenes, cells were treated with Dox in a chemically defined medium on alternate days of the differentiation protocol. FACS analysis showed consistent Dox-induced expression of TRPC6-EGFP throughout the 14 days of the differentiation protocol (Figure 2C). Immunostaining showed expression of the podocyte markers podocalyxin (PODXL1), synaptopodin (SYNPO) and podocin (NPHS2) (Figure 2D).

**Figure 3:**
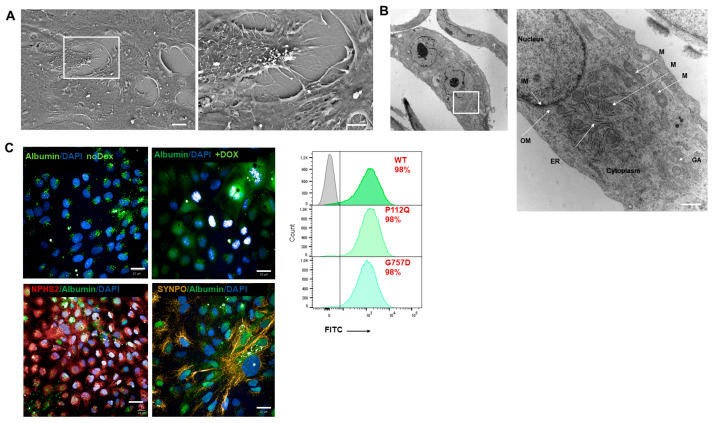
Human iPSC—TRPC6-derived ipodocytes manifest primary and secondary cell processes and albumin endocytosis on day 14 of differentiation. (**A**) Scanning electron microscopy (SEM) images. Scale bar = 2 µm. (**B**) Transmission electron microscopy (TEM) images. (IM) Inner membrane; (OM) outer membrane; (ER) endoplasmic Reticulum; (M) mitochondria; and (GA) Golgi apparatus. Scale bar = 1000 nm. (**C**) Functionality of podocytes, above panel, differentiating no Dox and Dox-induced endocytosing Albumin–FITC; bottom panel, denoting Nephrin (NPHS1) (red) and Synaptopodin (SYNPO) (yellow) endocytosing Albumin–FITC. Scale bar 20 µm. Flow cytometry analysis of albumin-positive iTRPC6 mutant podocytes. Gray population (left side) of box indicates podocyte population without albumin uptake and green podocyte population (right side) indicates albumin intake in TRPC6-WT, P112Q and G757D podocytes.

Furthermore, the ultra-structure of day 14 podocytes was visualized by scanning electron microscopy (SEM) and transmission electron microscopy (TEM). SEM showed the development of primary and secondary foot processes while the neighboring interdigital processes are connected by molecular complexes known as slit diaphragms (Figure 3A) [44]. In TEM, podocytes showed a well-developed Golgi apparatus, abundant endoplasmic reticulum, and many mitochondria. Podocytes contain many large lysosomes of different stages at high numbers in the cytoplasm, which are responsible for catabolizing proteins such as albumin taken up from the glomerular filtrate (Figure 3B). In addition, the functionality of podocytes is demonstrated by their endocytosis of Albumin–FITC (Figure 3C). The expression of the respective mutant and wild-type TRPC6-GFP throughout the differentiation protocol is shown in Figure S4. GFP expression in iPSCs and earlier progenitors is relatively homogenous and strong (around 90%), but as the cells mature, the heterogeneity in cell composition increases, and a mosaic expression of the TRPC6-GFP in both mutants and wild-type can be observed, with the lowest level seen in GoF mutants. TRPC6 protein on day 14 can be analyzed by immunoblotting Figure S2. 

**Figure 4:**
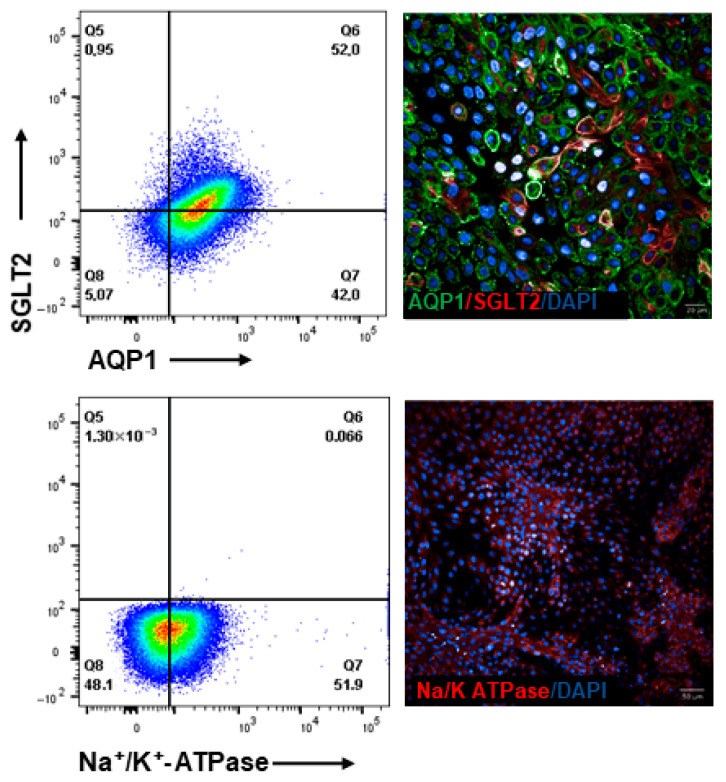
Efficiency of hiPSC iTRPC6-derived Proximal tubular epithelial cells (PTEC). Flow cytometry analysis and fluorescence microscopy of cells on day 16 post-differentiation induction for AQP1, SGLT2, and Na^+^/K^+^-ATPase. Scale bar 20 µm and 50 µm.

To further explore the potential of the GSH platform for the forward programming of hiPSCs into renal progenitor cells, we focused on generating proximal–tubule epithelial-like cells (PTEC) [37,40]. The day 8 renal vesicle-like cells were harvested and plated as single cells at high density in the presence of renal epithelium growth medium (REGM). After 6 days of continuous culture in REGM, the cells appear heterogeneous with several tubular epithelial cells (Figure 4). The expression of proximal tubular markers like Na^+^/K^+^-ATPase, AQP1, and SGLT2 was noteworthy by day 14 (Figure 4 and Figure S3A) and validated a PTEC phenotype of these cells.

### 3.3. Calcium (Ca^2+^) Influx Measurements iTRPC6-Derived Podocytes

**Figure 5:**
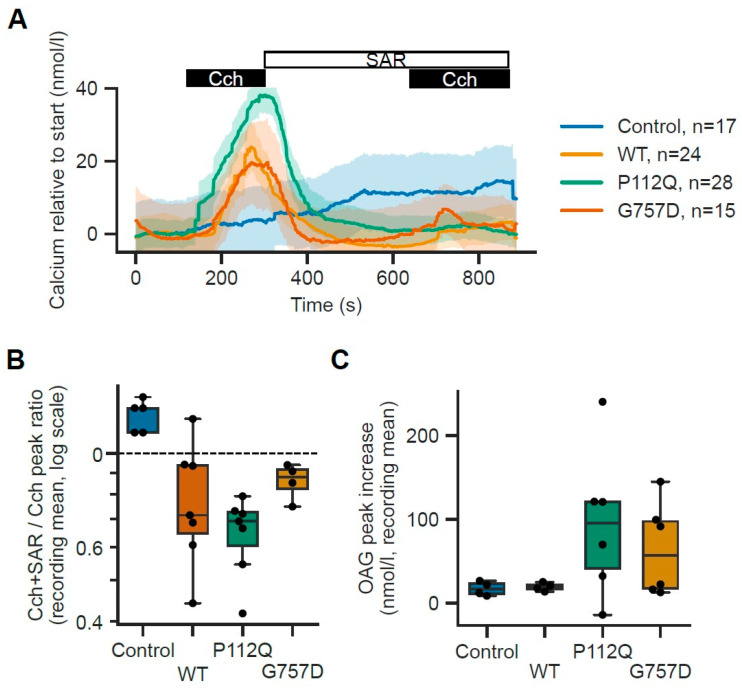
Functional characterizations of disease-related TRPC6 mutations by calcium influx measurement. Changes in intracellular calcium concentration were measured in Fura-2 AM-loaded ipodocytes expressing TRPC6 wild-type (WT), GoF (P112Q) and LoF (G757D) treated with doxycycline (0.5 µg/mL) 24 h before measurement and control cells with no genetic modification. (**A**) Carbachol (Cch: 100 µM): Cch was added about 2 min after the start of the measurement for about 3 min. After these 5 min, solution containing the TRPC6 blocker SAR7334 (SAR; 100 µM) was applied for 5 min, followed by another stimulation using Cch in the presence of SAR. Traces show the mean ± s.e.m. for the indicated number of cells. (**B**) Ratio of the Cch-induced peak in the presence of SAR7334 (SAR; 100 nM) compared to the initial stimulation in the absence of SAR. The box plot graph (box: 25–75 percentile; horizontal line is the median; whiskers represent 1.5× IQR) shows differences in the amplitude of the responses from controls, TRPC6 wild-type-, P112Q-, and G757D-expressing podocytes. The black dots are the individual average values for each individual recording. Statistical analysis was performed using a Mann–Whitney-U test on the averages of all cells within a single recording, followed by Benjamini–Hochberg correction for multiple comparisons. (**C**) In a separate experiment, the TRPC6 activator DOG (100 µM) was added about 3 min after the start of the measurement for about 5 min. Peak increases in intracellular Ca^2+^ are plotted as box plots (box: 25–75 percentile; horizontal line is the median; whiskers represent 1.5× IQR). Statistical analysis was performed using a Mann–Whitney-U test on the averages of all cells within a single recording, followed by Benjamini–Hochberg correction for multiple comparisons. (OAG peak G757D vs. WT *p*-value = 0.634921; OAG peak P112Q vs. WT *p*-value = 0.634921; SAR ratio G757D vs. control *p*-value = 0.038902; SAR ratio P112Q vs. control *p*-value = 0.034088; SAR ratio WT vs. control *p*-value = 0.038902; SAR ratio G757D vs. WT *p*-value = 0.648485; SAR ratio P112Q vs. WT *p*-value = 0.634921).

The iPSC-derived ipodocyte model was used to measure the TRPC6 activity in response to carbachol, an activator of the TRP channel. iTRPC6-GoF showed increased signs of Ca^2+^ influx compared to the iTRPC6-WT and control cell lines (CAMi014-A, with no transgenic integration) whereas TRPC6-LoF cells showed decreased Ca^2+^ influx in comparison to wild-type and GoF (Figure 5A). Since Cch stimulation may not be specific to the activation of TRPC6 channels; we treated cells with the specific inhibitor SAR7334 (SAR), followed by a second stimulation using Cch. The difference in the elicited amplitudes correlates with the function of the studied TRPC6 constructs (Figure 5B). Transfection with either WT, P112Q or G757D resulted in significantly larger TRPC6-specific Ca^2+^ influx (OAG peak G757D vs. control *p* value = 0.205714; OAG peak P112Q vs. control *p* value = 0.171429; OAG peak WT vs. control *p*-value= 0.885714) to controls. Furthermore, in separate experiments, we measured Ca^2+^ influx in response to 1,2-dioctanoyl-sn-glycerol (DOG), a direct TRPC6 channel activator. iTRPC6GoF showed a trend towards an increase in Ca^2+^ influx in comparison to TRPC6-WT but with very large variability. However, similar observations could be made for LoF-expressing cells which also showed a higher Ca^2+^ influx in comparison to TRPC6-WT (Figure 5C). This suggests that the cell may upregulate other TRPC channels (like TRPC1 or TRPC3) or Orai1/STIM1 (Store-Operated Calcium Entry) to compensate for the TRPC6 deficit.

In addition, Ca^2+^-influx in iTRPC6-WT, GoF, and LoF mutants was measured upon addition of the TRPC6 channel inhibitor, SAR7334 [45]. Treating TRPC6-expressing cells with SAR7334 showed no significant response to carbachol-induced Ca^2+^ influx and no signal amplitude to DOG-induced Ca^2+^ influx in fura-2-loaded cells (Figure 5A,B), indicating that the antagonist blocked the TRPC6 channel activity in the ipodocytes.

## 4. Discussion

TRPC6 channels have been implied to play a role in the progression of sporadic and familial forms of FSGS [1]. Distinct GoF and LoF mutations in *TRPC6* were described in different families and individuals, all showing a dominant mode of inheritance, typically with late onset of disease [15,16,18,23]. To date, the identification and characterization of *TRPC6* GoF and LoF mutants have suggested that both result in the same phenotype despite opposite effects on calcium influx [22,23,28]. However, we recently identified a *TRCP6* LoF variant resulting in a truncated TRPC6 protein with a dominant negative activity on calcium influx, although family members carrying this mutation did not develop FSGS [46]. These findings highlight the need for an experimental system to allow for a controlled investigation of the role of TRPC6 GoF and LoF mutations in FSGS at the cellular level.

In this study, we report the generation of a human iPSC-derived FSGS model by overexpressing the wild-type and two mutant forms of TRPC6 calcium channel proteins in ipodocytes. Ca^2+^ influx imaging on ipodocytes expressing the GoF (P112Q) and LoF (G757D) mutants showed increased and decreased calcium activities consistent with a GoF and LoF phenotype, respectively. These findings demonstrate that the model reliably recapitulates the expected channel-specific calcium signaling phenotypes in a human podocyte context.

However, a paradoxical increase in DOG-induced calcium influx observed in the G757D LoF mutant (Figure 5C) in comparison to WT suggests a complex compensatory landscape within the podocyte. A similar hyper-response has been documented in other ion channelopathies, where the loss of a primary conductance leads to the upregulation of synergistic channels, such as TRPC3 or Orai1, to preserve homeostatic calcium signaling [22]. Furthermore, TRPC6 is known to form heterotetrameric complexes with TRPC1 and TRPC3; the G757D mutation may alter the stoichiometry of these complexes, leading to a pool of channels that are less active at baseline but more sensitive to lipid-mediated activation [14,22,45]. This observation reinforces the utility of our hiPSC model in capturing not just the primary channel defect, but the integrated cellular adaptation and compensation that occur in response to chronic TRPC6 dysfunction.

However, whether the overexpression of these genes and differential intracellular calcium levels also result in phenotypical podocyte effacement still needs to be elucidated. Our study successfully profiles the functional calcium-handling of isogenic hiPSC-derived GoF and LoF lines, revealing the key differences in the downstream pathways driven by TRPC6 dysregulation. Further differences, such as cytoskeletal hyper-contractility and endoplasmic reticulum stress via chronic calcium overload [47,48] driven by GoF mutations or a failure of mechanotransduction and a lack of pro-survival signaling due to lower calcium influx resulting from LoF mutations [22,49], need to be studied and confirmed to characterize the respective mutational cell fates. Future studies leveraging these cell lines and detailed subcellular characterization will be instrumental in establishing the therapeutic window of TRPC6 in kidney disorders.

A hallmark of podocyte injury is foot process effacement (FPE), a dynamic process that involves actin reorganization and the disintegration of the slit diaphragm. Previous work has shown that dysregulated TRPC6-mediated Ca^2+^ signaling acts as an upstream trigger for the remodeling of the podocyte cytoskeleton. Elevated intracellular Ca^2+^ levels downstream of TRPC6 activation can activate actin-regulating pathways, including RhoA/ROCK signaling and the calpain-mediated cleavage of focal adhesion components, leading to disruption of the podocyte actin cytoskeleton and the disappearance of foot processes [15,50]. The absence of a 3D architectural environment and physiological shear stress in culture conditions may obscure the actual extent of structural remodeling, given the mechano-sensitivity of TRPC6. Previous animal models have not identified the critical threshold of cell stress—the point at which podocyte structural changes trigger pathways leading to irreversible podocyte injury. Experimental platforms that recapitulate the glomerulus in vitro environment, especially the basement membrane and the interaction between podocytic foot processes and endothelial cells, will enable the investigation of the pathophysiological effects of these mutations [51,52]. The established TRCP6-WT, GoF and LoF iPSC lines allow for the generation of both endothelial cells and ipodocytes to study the role of TRPC6-dependent calcium modulation in such a platform.

Our model represents a significant methodological advance from early hiPSC-podocyte studies. While foundational work by Kim et al. (2017) [53] established robust protocols for differentiating hiPSCs into podocyte-like cells expressing key markers such as Nephrin and Podocin, those models primarily utilized standard, non-isogenic cell lines. This limits the ability to attribute phenotypic changes solely to a single genetic variant, as inter-individual genetic variation can confound cellular responses. Furthermore, while Musah et al. (2018) [54] advanced the field by introducing ‘Glomerulus-on-a-Chip’ technology to provide the mechanical cues (shear stress and cyclic stretch) necessary for podocyte maturation, their focus was on structural assembly and filtration barrier function. Our system complements these architectural advancements by providing a genetically precise, inducible switch for TRPC6 activity which can be activated temporally, avoiding the undesirable effect of the mutation in early stages of development and lineage specification.

The applied differentiation protocol allows for rapid generation podocytes, along with multiple renal cell types and the ability to analyze them at different time points during the differentiation process reflecting the different developmental stages [36]. Thus, it provides opportunities for studying both early- and late-onset FSGS and other kidney diseases, such as congenital nephrotic syndrome and steroid-resistant nephrotic syndrome [52,55]. By activating the genetic switch only after the cells have reached a mature podocyte-like state, we can simulate the transition from healthy glomerular function to pathological calcium signaling more accurately, with a level of control unavailable in the earlier patient-derived or constitutive hiPSC models. Interestingly, the used Tet-On dual GSH targeting system [39] showed stable expression of intact i*TRPC6*-wt-, GoF and LoF mutant proteins in iPSC. Upon induction of differentiation and maturation, expression levels remained constant until day 8 of podocyte induction and declined to 70% on day 14 (Figure S3). The translational value of our inducible isogenic hiPSC model extends beyond simple disease modeling to encompass the fields of drug discovery and precision nephrology. By providing a stable, purifiable, homogeneous population of human podocytes, this platform is ideally suited for high-throughput screening (HTS) to identify novel TRPC6 modulators. Unlike traditional heterologous systems (e.g., HEK293 cells), our model captures the cell-specific signaling context of the podocyte, including its unique cytoskeletal and ER stress responses. Furthermore, this system serves as a ‘genetic chassis’ for personalized medicine; by introducing patient-specific mutations into this controlled background, clinicians can unambiguously determine the functional effects of novel *TRPC6* variants. Such a trial-in-a-dish approach could prioritize patients for TRPC6-targeted therapies and ensure that only those with a confirmed gain-of-function profile receive effective inhibitors, thereby reducing the risk of adverse effects associated with excessive TRPC6 suppression.

TRP channels are ubiquitously expressed in various cell types of the cardiovascular system. TRPC6 has been detected in the sinoatrial nodal cells and in cardiomyocytes [56,57]. To demonstrate broader application of the generated iTRPC6-EGFP cell lines beyond kidney cell types, they were also differentiated into cardiomyocytes using an established protocol [41]. The beating cardiomyocytes on day 8 were assessed by flow cytometry and showed around 95% cardiac troponin (cTNT) marker (Figure S3B). This highlights the applicability and versatility of these engineered TRPC6-iPSC lines to be used in other cell types of interest where TRPC6-mediated signaling or disease mechanisms need to be investigated.

In summary, we produced isogenic human iPSC lines that differ only in their specific *TRPC6* mutations and then differentiated them into kidney podocytes to examine mutation-dependent calcium signaling and facilitate the mechanistic changes associated with TRPC6-mediated FSGS. While two-dimensional podocytes derived from iPS cells provide a robust platform for investigating TRPC6-mediated signaling, their integration into three-dimensional renal organoids or micro-physiological systems could further enhance the ability to capture structural aspects of podocyte injury. The cell lines established here, therefore, provide a versatile human platform for investigating TRPC6-driven pathomechanisms and for evaluating therapeutic strategies in various TRPC6-expressing tissues.

## 5. Conclusions

We developed an hiPSC-based system that allows for controlled the overexpression of *TRPC6* wild-type and disease-associated variants, and showed that these cells can be reliably differentiated into functional podocytes. The calcium signaling responses we observed reflect the expected gain- and loss-of-function effects of the mutations, supporting the validity of this model. Importantly, this approach offers a more relevant alternative to traditional animal models, which often fail to capture key aspects of TRPC6-associated FSGS. By maintaining native cellular interactions and offering scalability, our platform creates new opportunities to study disease mechanisms in a more physiologically meaningful setting. Ultimately, these iTRPC6 lines may serve as a useful tool for inestigating TRPC6-related pathology across different tissues and for developing more targeted therapeutic strategies.

## Data Availability

The original contributions present in this study are included in the article/Supplementary Material. Further inquiries can be directed to the corresponding author.

## Supplementary Materials

The following supporting information can be downloaded at: https://www.mdpi.com/article/10.3390/cells15080712/s1, Figure S1: (A) Genotyping results for AAVS1-TRE-TRPC6-EGFP targeted hiPSCs. (B) Homozygous (HOM) lines for each allele indicate successfully targeted alleles of the AAVS1 loci. (C) Characterization of hiPSC lines expressing pluripotency markers OCT4, SOX2, and SSEEA. Scale bar 100 µm (D) Embryoid bodies were formed to check the pluripotency differentiation potential of iTRPC6 mutants (WT, P112Q, and G757D) and RT-PCR confirmed the differentiation of each line into endodermal (Goosecoid (GSC), Sox17), mesodermal (BrachyuryT and MIXL1), and ectodermal cells (NESTIN and NEUROD1). Scale bar 100 µm. Figure S2: Detection of TRPC6 protein extracted from hiPSCs by Immunoblotting. (A) iTRPC6 wild-type (WT), GoF (P112Q) and LoF (G757D) protein expression on day 0. (B) iTRPC6 wild-type (WT) GoF (P112Q), and LoF (G757D) protein expression on day 14 the of the differentiation protocol. Control is the protein extracted from cells without genetic modification. Figure S3: Efficiency of iPSC-derived PTEC. (A) Flow cytometry analysis of iTRPC6 wild-type (WT), GoF (P112Q) and LoF (G757D) iTRPC6 expressing cells on day 16 post-differentiation induction for AQP1, SGLT2, and Na^+^/K^+^-ATPase. (B) Flow cytometry analysis and fluorescence microscopy of cells on day 8 post-differentiation, demonstrating the induction of the cTNT marker. Scale bar 100 µm. Figure S4: Differential expression of iTRPC6-wt, GoF and LoF cell lines, indicating GFP expression throughout the differentiation process. GFP was induced on day 0 and indicated TRPC6 expression around 90% until day 8. A reduced expression was observed on day 14 at around 70%. FACs analysis were performed on alternative days from day 0 until day 14. The gray color (left side in each boxplot) represents untransfected population of cells while green color (right side in each box plot) represents TRPC6-GFP tagged population of podocytes. Supplementary Table S1: primer sequences. Supplementary Table S2: Cell culture media formulations, Supplementary Table S3: Primary antibodies.
