# Supplementary material for: An Inducible hiPSC-Derived Human Podocyte Model for Functional Analysis of TRPC6 Variants Associated with FSGS
Source: Cells. 2026 Apr 17;15(8):712. doi: 10.3390/cells15080712 (PMC13115377; doi:10.3390/cells15080712)
Supplement: Supplementary file 1 [file cells-15-00712-s001.zip › cells-4240490-supplementary.pdf]

Supplementary Figures

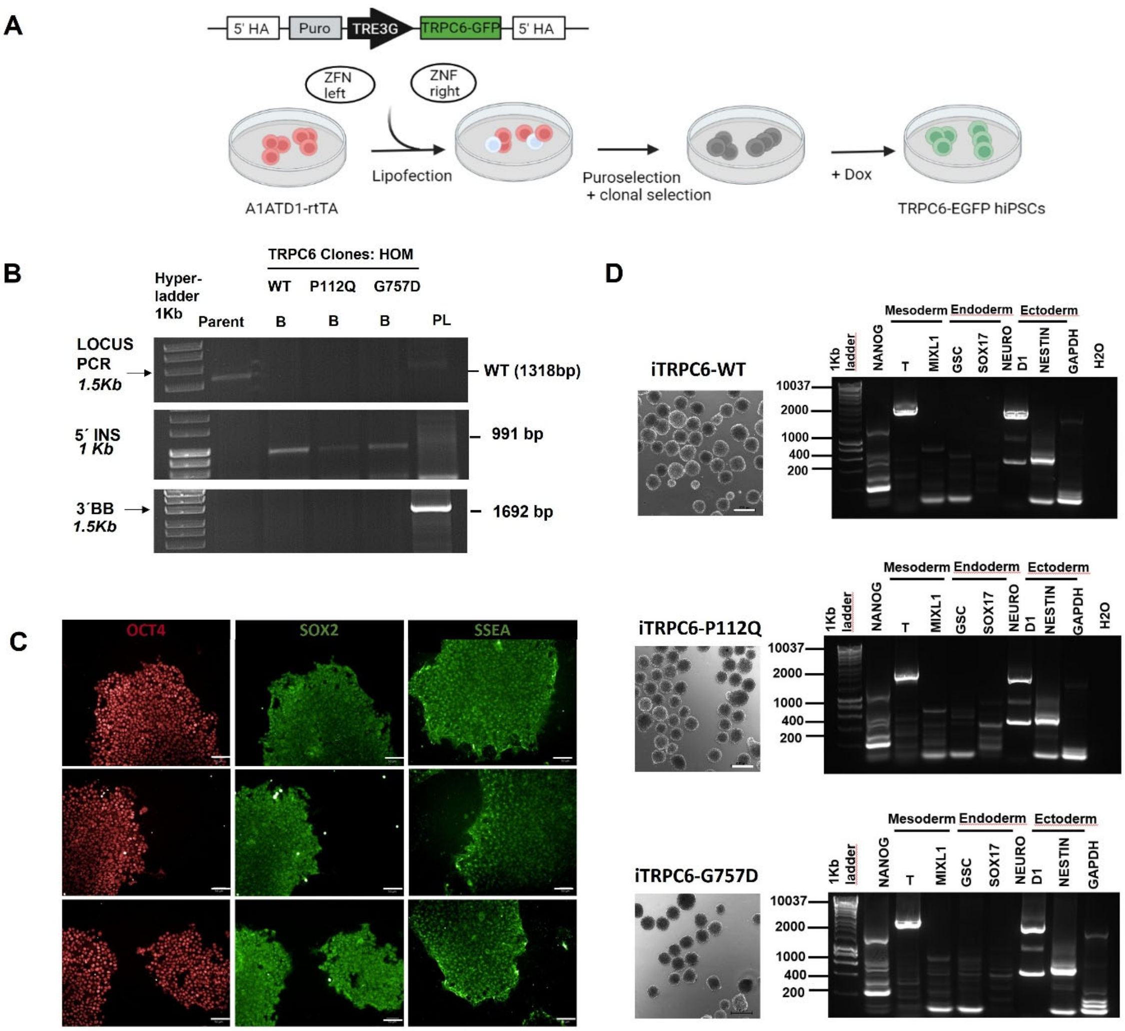

Figure S1: (A) Genotyping results for AAVS1-TRE-TRPC6-EGFP targeted hiPSCs. (B) Homozygous (HOM) lines for each allele indicate successfully targeted alleles of the AAVS1 loci. (C) Characterization of hiPSC lines expressing pluripotency markers OCT4, SOX2, and SSEEA. Scale bar 100 μm (D) Embryoid bodies were formed to check the pluripotency differentiation potential of iTRPC6 mutants (WT, P112Q, and G757D) and RT-PCR confirmed the differentiation of each line into endodermal (Goosecoid (GSC), Sox17), mesodermal (BrachyuryT and MIXL1), and ectodermal cells (NESTIN and NEURODl). Scale bar 100 μm.

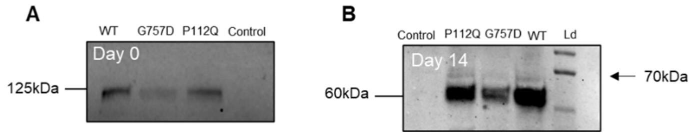

Figure S2: Detection of TRPC6 protein extracted from hiPSCs by Immunoblotting. (A) iTRPC6 wild-type (WT), GoF (P112Q) and LoF (G757D) protein expression on day 0. (B) iTRPC6 wild-type (WT) GoF (P112Q), and LoF (G757D) protein expression on day 14 the of the differentiation protocol. Control is the protein extracted from cells without genetic modification.

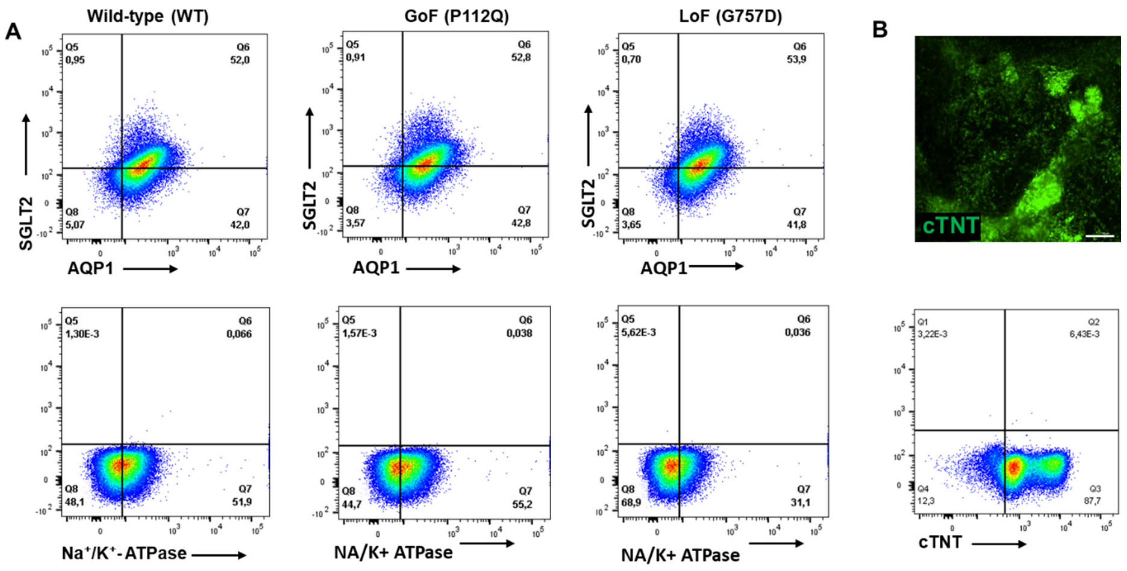

Figure S3: Efficiency of iPSC-derived PTEC. (A) Flow cytometry analysis of iTRPC6 wild-type (WT), GoF (P112Q) and LoF (G757D) iTRPC6 expressing cells on day 16 post-differentiation induction for AQP1, SGLT2, and Na^{+}/K^{+}-ATPase. (B) Flow cytometry analysis and fluorescence microscopy of cells on day 8 post-differentiation, demonstrating the induction of the cTNT marker. Scale bar 100 µm.

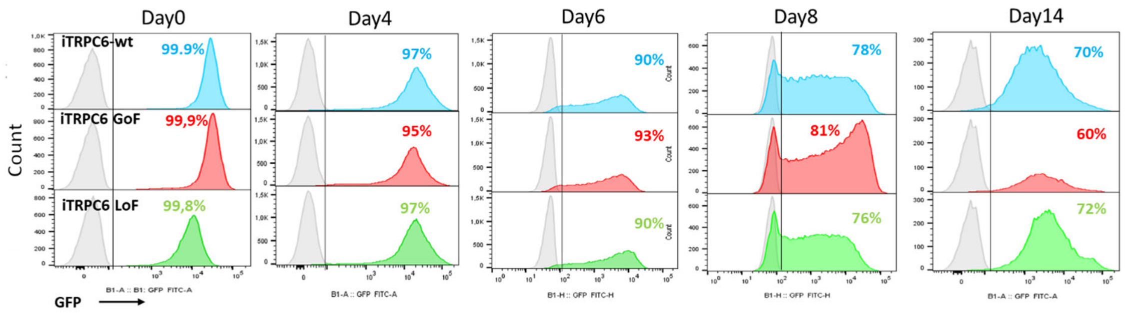

Figure S4: Differential expression of iTRPC6-wt, GoF and LoF cell lines, indicating GFP expression throughout the differentiation process. GFP was induced on day 0 and indicated TRPC6 expression around 90% until day 8. A reduced expression was observed on day 14 at around 70%. FACs analysis were performed on alternative days from day 0 until day 14. The gray color (left side in each boxplot) represent untransfected population of cells while green color (right side in each box plot) represent TRPC6-GFP tagged population of podocytes.

Supplementary Table S1. primer sequences

Primer name	Sequence 5‘→3‘
**Cloning**	
TRPC6-SpeI-fw	CGCCACTAGTATGAGCCAGAGCCCGGC
TRPC6-EcoRI-rv	TCACGAATTCTCTATTGGTTTCCTCT
eGFP-EcoRI-fw	CACCGAATTCGTGAGCAAGGGCGAGGA
eGFP-KpnI-rv	ATTCGGTACCTACTTGTACAGCTCGTCCATGC
**Sequencing**	
TRPC6-WT-seq-fwd	GTGATCGCTCCACAAGCCTAT
TRPC6-112-seq-fwd	TGATCGCTCCACAAGCCTATC
TRPC6-757-seq-fwd	CTACTCCTACTACATTGGTGC
SV40-pA-R-156-175-seq	GAAATTTGTGATGCTATTGC
PUC-M13-F-seq	CCCAGTCACGACGTTGTAAAACG
PUC-M13-R-seq	AGCGGATAACAATTTCACACAGG

PCR name	Primer binding sire	Sequence 5‘→3‘
Locus PCR	hAAVSI-genome (5’)	CTGTTTCCCCTTCCCAGGCAGGTCC
	hAAVSI-genome (3’)	TGCAGGGGAACGGGGCTCAGTCTGA
5'-INT PCR	hAAVSI-genome (5’)	CTGTTTCCCCTTCCCAGGCAGGTCC
	Puro-N-ter Rv	TCGTCGCGGGTGGCGAGGCGCACCG
3'-INT PCR	TRPC6-GFP-C terminal	GTGATCGCTCCACAAGCCTAT
	hAAVSI-genome (3’)	TGCAGGGGAACGGGGCTCAGTCTGA
3'-BB PCR	TRPC6-GFP-C terminal	GTGATCGCTCCACAAGCCTAT
	pUC-AAVS1- vector BB (3')	ATGCTTCCGGCTCGTATGTT

Supplementary Table S2. Cell culture media formulations

Media	Composition
Basal medium	RPMI-1640 and B27 minus insulin supplement (1x)
Cardiac priming medium	Basal medium + 6μM CHIR 99021
Cardiac induction medium	Basal medium + IWP-2
CM maintenance medium	RPMI 1640 plus B-27 supplement (1x)

Supplementary Table S3. Primary antibodies

Name	Species	Company
PODXL	Mouse monoclonal	R&D System

SYNPO	Mouse monoclonal	Progen
WT1	Rabbit polyclonal	Santa Cruz
NPHS2	Rabbit polyclonal	Abcam
SIX2	Mouse monoclonal	Abnova (tebubio)
PAX2	Rabbit polyclonal	Invitrogen
HOXB7	Mouse monoclonal	R&D System
AQP1	Rabbit polyclonal	Proteintech
Na+/+K ATPase	Rabbit Polyclonal	Abcam
SGLT2	Mouse monoclonal	Abcam
